# Does COVID‐19 really impact on the oxy‐hemoglobin dissociation curve?

**DOI:** 10.1002/jha2.126

**Published:** 2020-10-31

**Authors:** Flávia Nóbrega, Vitor Augusto Queiroz Mauad, Davimar Miranda Maciel Borducchi

**Affiliations:** ^1^ Faculdade de Medicina do ABC Santo Andre Brazil

## Abstract

The COVID‐19 pandemic has had a crucial impact on lifestyle worldwide. In this way, many studies have been presented, leading to continuous revaluation and questioning of conducts and concepts. Such is the case of the Chinese study suggesting that the new coronavirus has the potential to code anomalous nonstructural proteins capable of dissociating the iron atom from the porphyrin structure, contributing significantly to the characteristic hypoxemia conditions of the disease. Considering the potential impacts of those findings, the current study aims to evaluate and measure the dissociation curve of oxy‐hemoglobin in COVID‐19 patients. The project consists of a retrospective cohort study with data regarding oximetry and hemoglobin levels collected from digital patients records. The correlation between the measures and estimated values by Spearmen test was 0.843 (*P* < .001). A multiple linear regression model was applied using measured SO2 as a predicted variable and hemoglobin, PO2, and pH levels as predictors. The coefficients were pH 0.16‐0.31 (*P* < .001); PO2 0.52‐0.66 (*P* < .001) and Hb 0.088‐0.059 (*P* = .706). Despite its limitations, the present study suggests that, at least in situations of clinical severity, the proposed mechanism does not appear to be universal or to have a significant clinical impact.

## 1 INTRODUCTION

The COVID‐19 pandemic has had a crucial impact on lifestyle worldwide. The emergence of an unpredicted respiratory syndrome, associated with a highly transmissible virus, required the adoption of strict social measures. In order to avoid an overload of the health system and the depletion of resources, the local government introduced social isolation measures and many economic activities were interrupted [1]. Therefore, the health crisis has also extended to the socioeconomic sector [2]. At the time of the present study, the number of infected people worldwide totalizes 37,450,150 cases, resulting in 1,077,218 deaths associated with the diagnosis. Brazil sums 5,091,000 cases and 150,326 deaths [3].

According to the World Health Organization's ethical guidelines for clinical research in the face of epidemics and pandemics, the development of instant and effective research data is considered a moral obligation, as well as protocols used to lead control strategies and mitigate effects [4]. In this way, many studies have been presented, leading to constant re‐evaluation and questioning of conducts and concepts. Among them, prepublished articles also stand out, suggesting theories that need further proof.

Such is the case of the Chinese study suggesting that the new coronavirus has the potential to code anomalous nonstructural proteins, capable of acting on the beta‐globin chains of hemoglobin, dissociating the iron atom from the porphyrin structure. Deoxyhemoglobin, being more sensitive to this attack, would generate a progressive reduction of the transporting gases specific chains. Therefore, oxygen transport capacity could be deficient and contribute significantly to the characteristic hypoxemia conditions of the disease [5]. Despite the fact that this is a nonpeer reviewed study and while it is true that the hypothesis is mostly believed not to be true by most virologists and hematologists, an in‐depth study of this hypothesis is still needed for adequate scientific rigor.

As the identification of the protein is laborious and the main point of interest is not whether or not it exists, but rather if it of meaningful clinical significance, or if it should be used to reconsider hemoterapic threshold, we developed this project consisting of a retrospective cohort study with data regarding oximetry and hemoglobin levels collected from digital patients records during the treatment of COVID‐19 to access the biological viability of this statement.

## 2 OBJECTIVE

The current study aims to evaluate and measure the dissociation curve of oxy‐hemoglobin in COVID‐19 patients in order to observe a possible disruption in the dissociation capacity beyond expected in those patients.

## 3 METHOD

### 3.1 Study design

It is a retrospective cohort study, with medical record data based on arterial blood gas parameters in patients with COVID‐19, who are typically subjected to this assessment twice a day. The data were collected from 30 patients.

### 3.2 Data tab

The data were collected from arterial blood gas analysis, in particular pH, PO2, and oxygen saturation. The oximetry values measured by Werfner's GEM 3500 hardware proceeds directly from the measurement of oxy‐hemoglobin and total hemoglobin and are not derived from mathematical models.

### 3.3 Inclusion criteria

In this study, we included patients diagnosed with COVID‐19 of 18 years old and above, who were treated for SARS‐COV2 at Hospital Estadual Mario Covas.

### 3.4 Forecasting mathematical analysis

The measured curve was compared with a mathematical forecast from Kelman's equation [6].

### 3.5 Statistical analysis

Once both standards were calculated and measured from each patient at the same time, the application of corrections in the quantitative model can be misleading, as the same variables affect both curves. Thus, the comparison was made directly by the Kolmogorov‐Smirnov method. We evaluated variables of interest in an exploratory way, comparing groups using the Student's *t* test or nonparametric variant when groups had <100 samples and the variable was not in a normal distribution.  Considering that the impact might be due to the binding of a viral product to hemoglobin and assuming a constant viral load, an exponential deterioration into lower hemoglobin levels would support the hypothesis. Therefore, hemoglobin levels can be correlated with directly measured SO2 levels.

The multiple regression model was used to predict SO2 by the variables of interest, such as Hb, pH, and PO2, in order to assess and confirm the individual impact of each component

## 4 RESULTS

**GRAPH 1:**
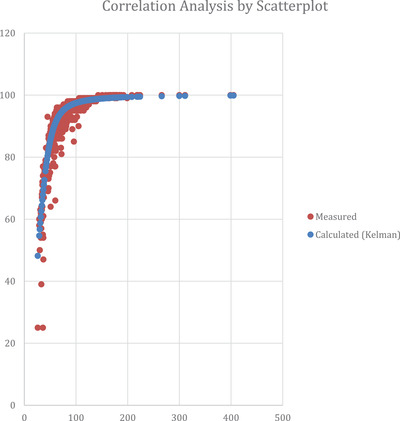
Scatterplot

**GRAPH 2:**
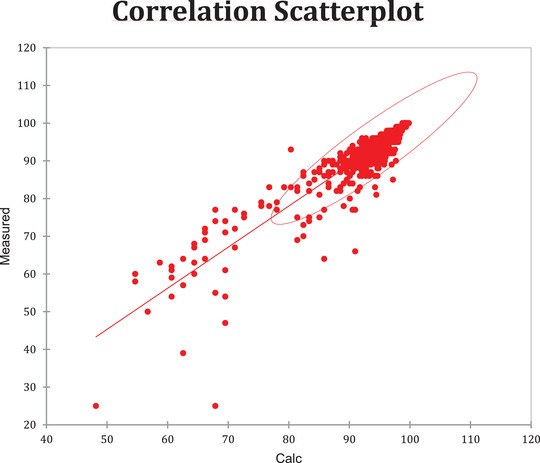
Spearman correlation analysis between measured and calculated SO2

A total of 30 patients were included, and 952 blood gas tests were performed. On average, each patient underwent 32 procedures (4‐91). The measured and estimated values were plotted on a scatterplot (Graph 1). The correspondence between both values was approached by the Spearman test, which resulted in 0.843 (*P* < .001), meaning a strong correlation (Graph 2). Hemoglobin levels were considered only by blood count and were collected simultaneously with blood gas collection. Only 470 blood samples had these data included. Hemoglobin levels range was 2.5‐14.5 mg/dL. A Kolmogorov‐Smirnof's curve comparison resulted in a significant *P* < 0.001.

**GRAPH 3:**
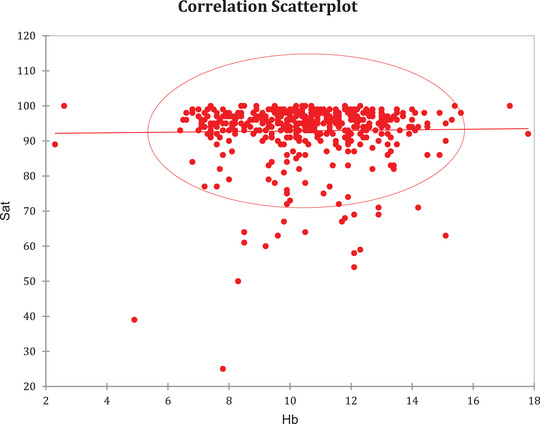
Correlation between measured SO2 and hematimetric levels

Assuming that the agent may have the potential to act on hemoglobin's oxygen‐binding capacity, the correlation between hemoglobin levels and the measured values of SO2 was analyzed (Graph 3) as a tool to rejecting the hypothesis.

Eventually, a multiple linear regression model was applied using measured SO2  as a predicted variable and hemoglobin, and PO2 and pH levels as predictors. The coefficients were pH 0.16‐0.31 (*P* < .001), PO2 0.52‐0.66 (*P* < .001), and Hb 0.088‐0.059 (*P* = .706).

## 5 DISCUSSION

Both the measured oxygen saturation and the calculated oxygen saturation demonstrated a high correlation (Graphs 1 and 2). However, a considerable discrepancy was observed in the measured curve, mainly at saturation levels below 70% (Graph 2), suggesting the presence of an unknown variable that interferes with hemoglobin's dissociation curve within the previously discussed ranges. It is an expected result given the known existence of possible disruptors in biological measurements.

Several factors can be related to this scenario.  Among the common disruptors, pH, carbon dioxide, and 2,3‐DPG [7, 8] play an elementary roll in decreasing hemoglobin's affinity for oxygen. The variation seen in hydrogen ions levels and carbon dioxide and their influence on hemoglobin saturation is called the Bohr effect [9]. The singular role of pH was effectively demonstrated in our sample (*P* < .001).

To evaluate the hypotheses produced by the mathematical model, we directly correlated hemoglobin levels to the results. The association between hematimetric levels and oximeter levels was found to be null (Graph 3). Therefore, low hemoglobin levels were not fundamental for low oxygen saturation, which is reinforced by the linear regression model. If the hypotheses were positive, lower levels of hemoglobin would have been expected to be linked to suppressed saturation levels, which were not observed. Thus, the study findings seem to refute the hypotheses of the direct impact of the Sars‐CoV‐2 virus on hemoglobin molecule functioning. However, it is important to emphasize that the analyzed sample was small (n = 30). It could consist of a study limitation because if the direct impact of the virus on hemoglobin was minimal or based on a random effect, this influence might not have been displayed. Furthermore, the group consisted of patients already with severe form of the disease, all coming from the Intensive Care Unit (ICU), so initial infection impacts could not be evaluated. Also, it is important to note the argument that there are more sensible methods to evaluate hemoglobin affinity, especially at the microcirculatory level; however, should any effect be present it seems to be either irrelevant or somehow mitigated, as it does not impact overall arterial oxygen levels.

Despite the limitations, the present study suggests that, at least in situations of clinical severity, the proposed mechanism does not appear to be universal or to have a significant clinical impact associated with it. Therefore, the initial hypotheses of a potential benefit in early blood transfusions for patients infected with COVID‐19 are refuted and, this study presents direct evidence to counterpoint said hypothesis, sending a sign that this should not be a focus in the current pandemic. Finally, we emphasize the importance of standard clinical evaluation for hemantimetric conduct in any patient and that COVID, despite not showing any specific effect here, does impact cardiac function [10] in at least a proportion of patients and that may impact transfusion thresholds.

As a take‐home message, one should note that, although the use of mathematical models serves as a helpful tool supporting interesting new proposals, it is essential that all proposals are always confirmed through biological models and cohorts studies before they are understood as potential clinical applications.
